# Comparison of methanol fixation versus cryopreservation of the placenta for metabolomics analysis

**DOI:** 10.1038/s41598-023-31287-3

**Published:** 2023-03-11

**Authors:** Jennifer K. Straughen, Alexandra R. Sitarik, A. Daniel Jones, Jia Li, Ghassan Allo, Carolyn Salafia, Andrea E. Cassidy-Bushrow, Nigel Paneth

**Affiliations:** 1 Department of Public Health Sciences, Henry Ford Health System, One Ford Place, Detroit, MI 48202 USA; 2 Department of Biochemistry & Molecular Biology and RTSF/MSU Mass Spectrometry and Metabolomics Core, Michigan State University, 603 Wilson Road, Biochemistry Room 215, East Lansing, MI USA; 3 Department of Pathology, Henry Ford Hospital, 2799 W. Grand Blvd, Detroit, MI 48202 USA; 4 Placental Analytics LLC, 187 Overlook Circle, New Rochelle, NY 10804 USA; 5 Departments of Epidemiology & Biostatistics and Pediatrics & Human Development, Michigan State University, 909 Fee Road, East Lansing, MI USA

**Keywords:** Metabolomics, Epidemiology

## Abstract

Methods for collection of placental tissue at room temperature for metabolic profiling are described. Specimens were excised from the maternal side of the placenta and immediately flash frozen or fixed and stored for 1, 6, 12, 24, or 48 h in 80% methanol. Untargeted metabolic profiling was performed on both the methanol-fixed tissue and the methanol extract. Data were analyzed using Gaussian generalized estimating equations, two sample t-tests with false discovery rate (FDR) corrections, and principal components analysis. Methanol-fixed tissue samples and methanol extracts had a similar number of metabolites (p = 0.45, p = 0.21 in positive vs. negative ion mode). In positive ion mode, when compared to flash frozen tissue, both the methanol extract and methanol-fixed tissue (6 h) had a higher number of metabolites detected (146 additional metabolites, p_FDR_ = 0.020; 149 additional metabolites, p_FDR_ = 0.017; respectively), but these associations were not found in negative ion mode (all p_FDR_ ≥ 0.05). Principle components analysis demonstrated separation of the metabolite features in the methanol extract, but similarity between methanol-fixed tissue and flash frozen tissue. These results show that placental tissue samples collected in 80% methanol at room temperature can yield similar metabolic data to flash frozen specimens.

## Introduction

Interest in incorporating placental functional measures and related information into human studies on the developmental origins of health and disease has increased in recent years and has expanded from measurements such as placental weight, thickness, and cotyledon number to epigenetics, magnetic resonance imaging, and metabolomics^1–4^. Placental metabolic processes in particular may offer insight into potential mechanisms linking suboptimal intrauterine exposures to childhood health outcomes and could also suggest therapeutic and nutritional interventions to mitigate the adverse effects of fetal programming on offspring. Despite the increased interest in measuring placental metabolism, few studies have incorporated these measures, which may be due, at least in part, to challenges involved in collecting specimens suitable for metabolic profiling. At present, placental metabolic profiling studies typically rely on flash freezing of specimens^5,6^. In many labor and delivery settings outside of research hospitals, maintaining cryopreservation capabilities for deliveries that can happen at any hour of any day is difficult and can be expensive. Thus, there is a need to develop lower cost methodologies that do not require specialized equipment.

Methanol is a well-described chemical fixative that acts by replacing water in tissue and has been used in the quenching step to halt enzymatic activity. In the cancer field, researchers have begun developing methods to generate metabolic profiles in tumor tissue by collecting tissue specimens in alternative fixatives, namely 80% methanol^7^. Previous work has noted that some metabolites remain in the tissue and some become suspended in the aqueous methanol^8^, which is advantageous when overcoming sampling issues due to a lack of availability of tissue (for example, from small core needle biopsies). As such, methodologies to date have primarily focused on capturing the metabolites from the aqueous methanol as opposed to both the aqueous methanol and the methanol-fixed tissue. Additionally, a few studies suggest the utility of collection of tissue specimens in 80% methanol at room temperature^7,9^.

These methods may have applications for placental metabolomics research. However, steps are needed to better understand how these methods might be applied to placental research. Previous work on collection of tissue in methanol for metabolomics has not established detailed protocols for collection of placental tissue nor have they optimized a methanol-based method for use with placental tissue. The goal of this manuscript is to compare placental metabolic profiles from samples collected in 80% methanol at room temperature to those collected from flash frozen specimens. In addition, the effect of varying the duration of fixation in 80% methanol on untargeted metabolomics profiles was examined.

## Methods

This study was approved by the Henry Ford Health System Institutional Review Board and the study was conducted in accordance with institutional regulations and guidelines. Waiver of written informed consent was granted for this study. The study utilized discarded tissue and all biospecimens and data are anonymous.

### Specimen collection

All placentas were sampled within 15 min after delivery of the placenta. After delivery of the placenta, the placenta was rinsed of nonadherent blood using phosphate-buffered saline and membranes were removed. Approximately 50-mg sections were excised from the maternal side of the placenta. Grossly abnormal regions were avoided and when possible, all sections were taken from the same cotyledon, but in all cases, samples were spatially confined to the same region of the placenta. After excising, sections were again rinsed in phosphate-buffered saline. To make comparisons with existing methods that rely on flash freezing, one specimen from each placenta was flash frozen in liquid nitrogen (also referred to as the gold standard). The remaining specimens were then immediately placed in high-performance liquid chromatography (LC) grade 80% methanol (~ 1.5 mL) in 2 mL polypropylene cyrogenic vials for a fixed amount of time at room temperature. The fixation time in methanol varied for each specimen (1, 6, 12, 24, and 48 h). After fixation in methanol for the predefined length of time, the tissue was removed from the methanol using stainless steel tweezers and both the methanol fixed tissue and the methanol extracts were placed at −80 °C for later analysis. All samples were collected in triplicate and 3 unique placentas were sampled. Finally, methanol controls for each placenta (no tissue added) were also prepared, which were used for quality assurance and removal of artifacts. In total, the analysis included 33 samples: 3 flash frozen samples (1 per placenta), 15 methanol-fixed placental tissue samples (1 sample for each of the 5 methanol fixation times, for each of the 3 placentas), and with the corresponding 15 methanol extract samples (1 sample for each of the 5 fixation times, for each of the 3 placentas).

Sample size was a priori determined based on reaching precision for the estimation of the integrative correlation coefficients. Simulations assuming that there are 300 common metabolites detected by both methods and for three placentas yielded standard errors for the integrative correlation coefficients of 0.006 to 0.018, which is reasonably small.

### Metabolic profiling

Untargeted metabolic profiling on the methanol-fixed placental samples and methanol extracts was performed by the Michigan Regional Comprehensive Metabolomics Resource Core Laboratory using LC/mass spectrometry (MS). Samples were kept on wet ice throughout the processing steps. For the assay processing requirements, a volume of 100 µL for liquid methanol extracts and an average weight of 49 (± 1) mg for solid placental tissue samples was measured. Methanol extracts and solid placental tissue samples were processed similarly. After adding 100 µL water to each sample, it was vortexed to mix. Samples were homogenized using a probe sonicator at 40% output power, 20% duty cycle for 20 s. Next, 900 µL of extraction solvent (8:2 methanol/water) with internal standards (Supplemental Table S1 online) was added to each sample. Samples were vortexed again and incubated at −20 °C overnight. After overnight incubation, samples were again vortexed. Protein precipitation was initiated by centrifugation at 4 °C for 10 min at 14,000 RPM. Supernatant from each sample was pooled to create a quality control sample. For the final LC/MS sample, approximately 250 µL (± 5 µL) of supernatant was used for tissue extracts and 200 µL supernatant was used for the methanol extract samples. For both types of samples, supernatant was transferred to an autosampler vial containing an insert and brought to complete dryness under a stream of nitrogen under ambient conditions. Samples were reconstituted with 100 µL of water: methanol (8:2 by volume). All samples were processed in random order.

### Optimized LC/MS analysis

Analysis was performed on an Agilent system consisting of an Infinity Lab II ultrahigh performance LC coupled with a 6545 QTOF mass spectrometer (Agilent Technologies, Santa Clara, CA) using a JetStream ESI source using the Waters Acquity HSS T3 column (2.1 × 50 mm, 1.8 µm; Waters Corporation, Milford, MA). Each sample was analyzed in both negative-ion and positive-ion modes. Mobile phase A consisted of 100% water with 0.1% formic acid whereas mobile phase B was 100% methanol with 0.1% formic acid. The gradient for both positive and negative ion modes was as follows: 0.2% (0 min), 75% (20 min), 98% (22 min), 98% (30 min), 2% (30.1 min) was used. The column was then reconditioned for 7 min with 2% before moving to the next injection. The flow rate was 0.46 mL/min and the column temperature was 40 °C. The injection volume for positive and negative mode was 5 µL and 8 µL, respectively. The following source parameters were used: drying gas temperature 350 °C, drying gas flow rate 10 L/min, nebulizer pressure 30 psi, sheath gas temp 350 °C and flow 11 L/min, and capillary voltage 3500 V, with internal reference mass correction.

Semi-quantitative data for known compounds was obtained by manual integration using Profinder v8.00 software (Agilent Technologies). Metabolites were identified by matching the retention time (± 0.1 min), mass (± 10 ppm) and isotope peak height and spacing to standards. For non-targeted feature mining, Agilent’s Find by Molecular Feature algorithm with recursion was used. A combined feature set was generated by merging untargeted features and named metabolites into a single feature list. The combined feature set underwent data reduction using *Binner*, which eliminates degenerate features including adduct, in-source fragment ions, and isotope peaks^10^. Metabolite features were annotated using RefMet^11^.

### Statistical methods

#### Additional data pre-processing

Positive and negative ion data were analyzed separately due to overlap in the detected metabolite features. A total of 6286 metabolite features in positive ion mode and 3810 metabolite features in negative ion mode were quantified across all samples and expressed as intensity values divided by tissue weight. Prior to statistical analysis, metabolite features with coefficient of variation/relative standard deviation (RSD) > 20% in the pooled QC samples were removed for quality control purposes, resulting in 3728 and 2777 total metabolite features in positive and negative ion mode, respectively. Some missingness was present in the data (on average, 9.9% of metabolite features were missing within each sample), which was primarily due to being below the limit of detection (i.e., data are missing not at random; left-censoring is present). Therefore, quantile regression imputation of left-censored data was performed, which has been shown to have the best performance for metabolomics data with this pattern of missingness^12^, using the *imputeLCMD* package in R (R Foundation, Vienna, Austria). Intensity values were log transformed prior to imputation, both for normalization purposes and to ensure positive values in the original scale. Following imputation, any metabolite features detected in at least one of the three blank methanol control samples were considered artifacts or contaminants and were removed prior to analysis, leaving 1413 metabolite features in positive ion mode and 1298 in negative ion mode. The use of methanol during extraction and storage is known to generate artifacts and this approach prevents erroneous interpretation of study results^13,14^.

#### Analysis

The number of metabolites detected (in positive and negative ion mode separately) among the methanol-fixed samples was modeled using a Gaussian generalized estimating equation (GEE) model, with sample type (tissue vs. methanol) and time (1, 6, 12, 24, and 48 h) as explanatory variables, and were clustered within placenta. Two sample t-tests with false discovery rate (FDR) Benjamini and Hochberg correction (p_FDR_ < 0.05 considered significant) was used to compare the number of metabolites detected in each experimental condition to flash frozen samples^15^. Because it is often difficult to summarize overall trends on high dimensional data without data reduction techniques, principal components analysis was performed on metabolites to capture variability in metabolomic profiles and to provide a graphical view of how the detected metabolites cluster by the experimental group (flash frozen, methanol extract, and methanol-fixed tissue). The top principal component(s) explaining a large percentage of the variability were retained for further analysis. This was determined by examining a scree plot to identify the number of components in which gain in variability explained was small thereafter. Variable loadings were used to examine the correlation between individual metabolites and principal components. GEE models were fit as described previously to principal components for methanol-fixed samples. Two sample t-tests with FDR correction were used to compare principal component differences with flash frozen samples. Additionally, GEE models were fit for individual metabolites, with FDR correction performed (p_FDR_ < 0.05 considered significant). Finally, 3-way Venn diagrams were used to assist with interpretation and visualization of the findings, more specifically to examine the proportion of metabolites in the flash frozen samples that were also in the methanol-extract and the methanol-fixed tissue samples. A metabolite was considered detected in each sample type if it was found in 2/3rds or more of samples (i.e., at least 2 out of the 3 total flash frozen samples, at least 10 out of the 15 total methanol-fixed tissue samples, and at least 10 out of the 15 total methanol extract samples).

## Results

### Comparison of number of metabolite features detected

**Figure 1:**
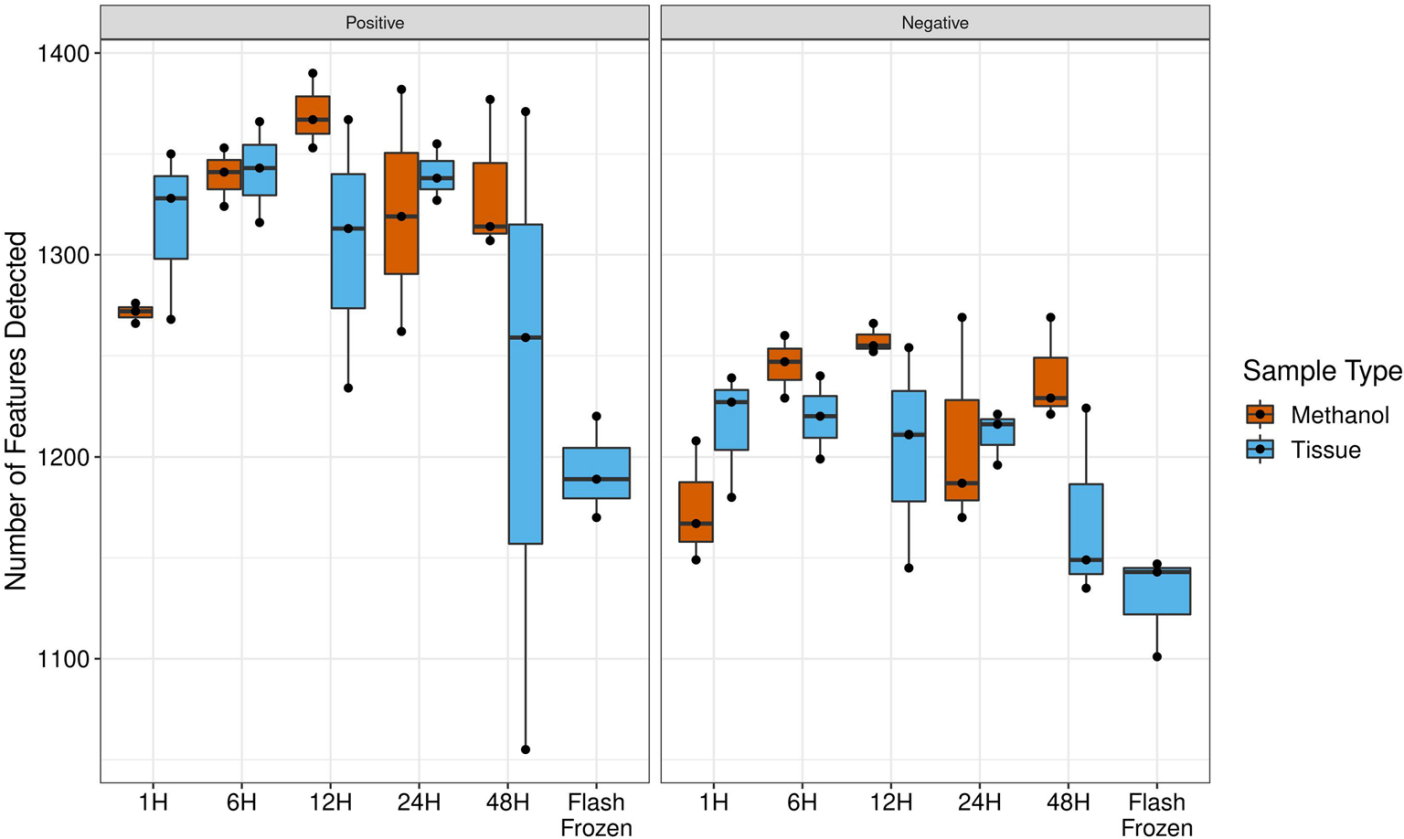
Number of metabolite features detected in positive and negative ion mode by sample type (tissue or methanol extract) and methanol fixation time. *H* hours.

On average, after removing potential contaminant or artifact metabolites, 1,305 metabolite features were detected in positive ion mode across the 33 samples [standard deviation (SD) = 72]; on average, fewer metabolite features were detected in negative ion mode [Mean (SD) = 1207 (45); Fig. 1]. The median RSD of all metabolites in positive ion mode was similar in methanol fixed tissue (median RSD [Q1, Q3] = 16.2% [9.1%, 28.3%]) and flash frozen tissue (median RSD [Q1, Q3] = 16.3% [9.2%, 28.4%]). Likewise, the median RSD in negative ion mode was similar in methanol fixed tissue (median RSD [Q1, Q3] = 11.1% [6.0%, 20.5%] and flash frozen tissue (median RSD [Q1, Q3] = 11.1% [6.0%, 20.6%]). In the fitted GEE model in positive ion mode, methanol-fixed tissue samples on average had 21 fewer metabolite features detected than the methanol extract (0.32 SDs), though this was not statistically significant (95% CI −75.6, 33.8; p = 0.45) and represents only a small fraction of detected metabolite features. A significant time effect for duration of fixation was not found. Specifically, for every 12 h of additional methanol fixation time, approximately nine fewer metabolite features were detected (0.13 SDs; 95% CI −27.3, 9.9; p = 0.36). Results were similar in negative ion mode, with a non-significant difference in the number of metabolite features detected comparing tissue vs. extract (β (95% CI) = -21 (−55, 12); p = 0.21) and by duration of fixation (β (95% CI) = −3 (−9, 3); p = 0.39). Both of these coefficients equated to relatively small effect sizes (0.55 SDs and 0.07 SDs, respectively). In positive ion mode, when compared to the flash frozen placental tissue, both the methanol extract and tissue samples fixed in methanol for 6 h had a significantly higher number of metabolite features detected (146 additional metabolite features, p_FDR_ = 0.020; 149 additional metabolite features, p_FDR_ = 0.017; respectively). Similarly, the methanol extract from the 12-h methanol fixation (177 additional metabolite features; p_FDR_ = 0.009) and tissue fixed in methanol for 24 h (147 additional metabolite features; p_FDR_ = 0.020) also had a higher number of metabolite features detected when compared to the flash frozen tissue samples. However, all other comparisons did not significantly differ (60% non-significant), including the number of metabolite features detected in 1-h methanol fixed tissue versus flash frozen tissue samples (122 additional metabolite features in 1-h tissue, p_FDR_ = 0.098). Further, when these comparisons were repeated in negative ion mode, no significant differences were found (all p_FDR_ ≥ 0.05), including a non-significant difference between the number of metabolite features detected in 1-h methanol fixed tissue and flash frozen tissue samples (85 additional metabolite features in 1-h tissue, p_FDR_ = 0.12).

### Principal components analysis of metabolite levels

**Figure 2:**
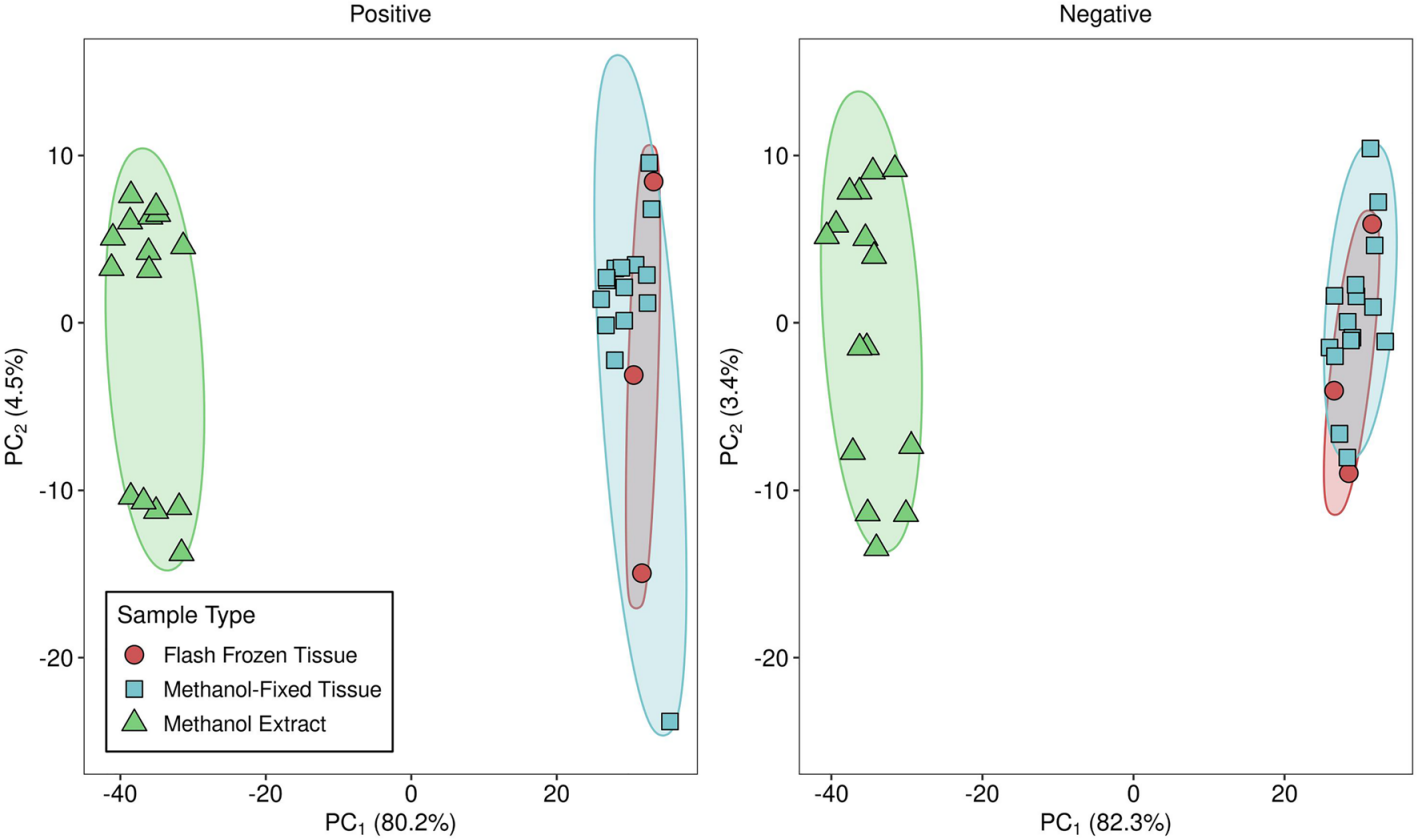
2-dimensional principal component plots stratified by positive and negative ion mode and by sample type (flash frozen tissue, methanol-fixed tissue, and methanol extract). Percentage of variability explained by each component is shown in parentheses on axis labels. *PC*_*1*_ first principal component, *PC*_*2*_ second principal component.

In the principal components analysis, the first component (PC_1_) explained 80.2% of the variability in metabolite levels in positive ion mode and 82.3% of the variability in negative ion mode; the second component (PC_2_) only explained 3.4% to 4.5% of the variability. The 2-dimensional PCA plot demonstrated a distinct metabolic profile of methanol extract samples, whereas flash frozen tissue and methanol-fixed tissue overlapped, indicating a relative similarity in metabolic profile (Fig. 2). PC_1_ was negatively correlated with all of the metabolite features in the positive ion data and all but one of the metabolite features in the negative ion data, meaning that higher values of PC_1_ indicates lower intensities of metabolite features in general. Given that both types of tissue samples had higher values of PC_1_, this therefore indicates that intensity levels for metabolite features were lower in tissue compared to methanol extract samples, which might be attributed to matrix suppression of ionization. The inverse was therefore taken for interpretation purposes in further testing so that higher values indicate higher values of metabolite features in general. When the inverse of PC_1_ was formally tested for differences using GEE models, a significant sample effect was detected in both positive and negative ion mode, where tissue samples had lower overall intensities (positive: β [95% CI] = −66.0 [−68.0, −64.0]; p < 0.001; negative: β [95% CI] = −64.5 [−66.0, −63.0]; p < 0.001). Further, additional fixation time resulted in increased intensity levels in negative ion mode (β [95% CI] = 0.46 [0.41, 0.51]; p < 0.001), but not in positive ion mode (β [95% CI] = 0.17 [−0.30, 0.64]; p = 0.47). The effect size was very large for the tissue effect (2 SDs lower in both positive and negative ion mode), while the time effect was small despite its significance in negative ion mode (1/100th of a SD in both cases). When compared to the flash frozen samples, levels of metabolites in all methanol-fixed tissue samples did not differ in both positive and negative ion mode (all p_FDR_ ≥ 0.49). However, all methanol extract samples significantly differed in their levels from flash frozen samples in both positive and negative ion mode (p_FDR_ < 0.05).

### Individual metabolite testing

**Figure 3:**
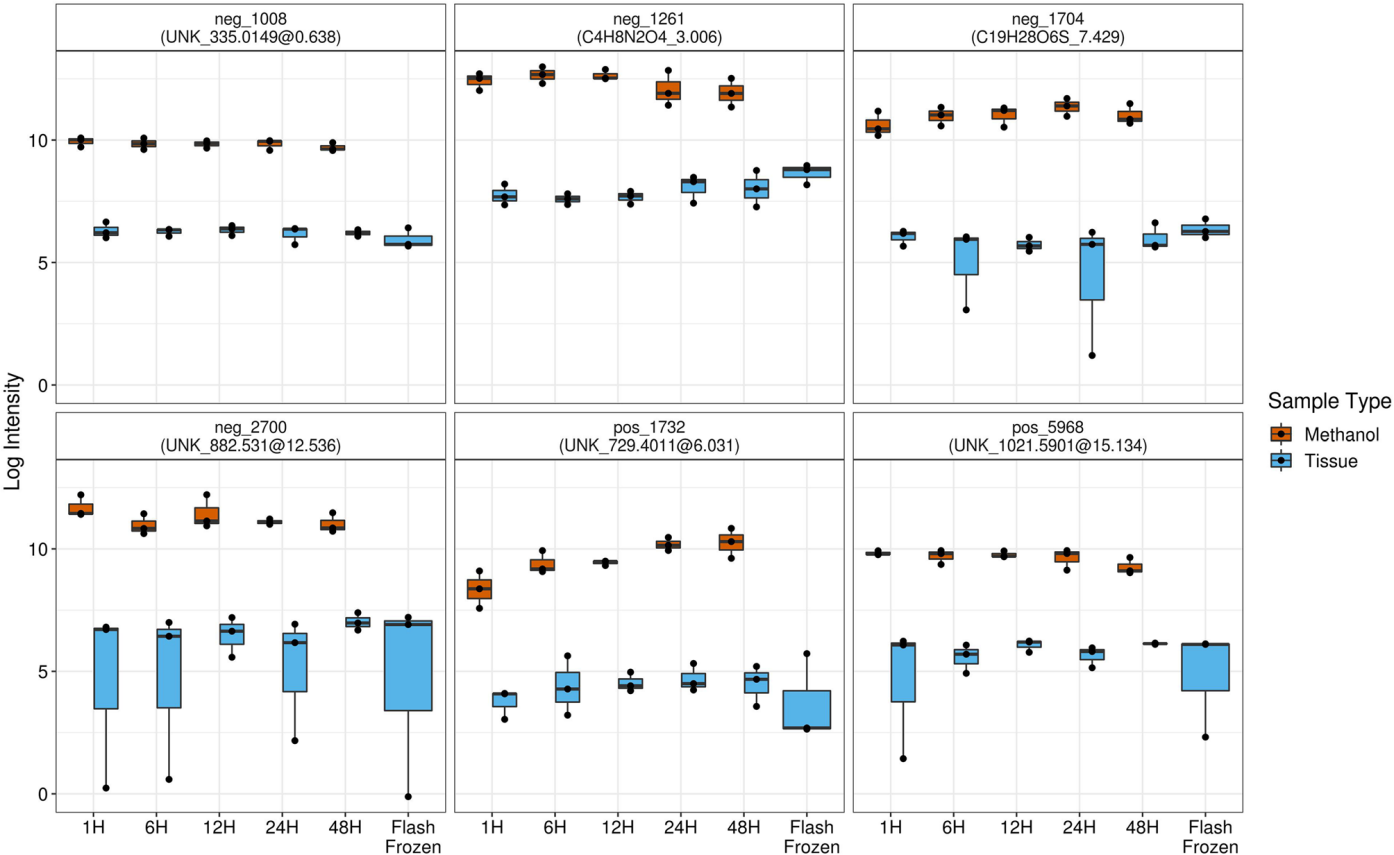
Six randomly selected metabolite features by sample type (tissue vs methanol extract) and methanol fixation time. *H* hours, *neg* negative, *pos* positive.

A sample of six individual, randomly-selected metabolites is shown in Fig. 3 for descriptive purposes. Consistent with the results of PC_1_, methanol-fixed tissue samples appeared to have much lower intensity values than the methanol extract. Flash frozen samples again appeared to behave similarly to methanol-fixed tissue samples. In GEE models (Supplemental Table S2 online), a significant sample type effect was detected for 2695 of the 2711 metabolite features across both polarities (99%), following FDR correction (all p_FDR_ < 0.05). Each of these significant coefficients was negative, signifying that the mean intensity of features in tissue samples was consistently significantly lower than methanol extract samples. A significant effect of duration of methanol fixation was detected for 573 (21%) metabolites (p_FDR_ < 0.05). Among these 573 metabolite features, 342 (60%) had a significantly increasing time effect, while 231 (40%) had a significantly decreasing time effect. The inconsistent direction of association with fixation time across features likely explains why the overall test using PC_1_ indicated a very small effect size in both positive and negative ion mode.

### Metabolite features shared across sample types

**Figure 4:**
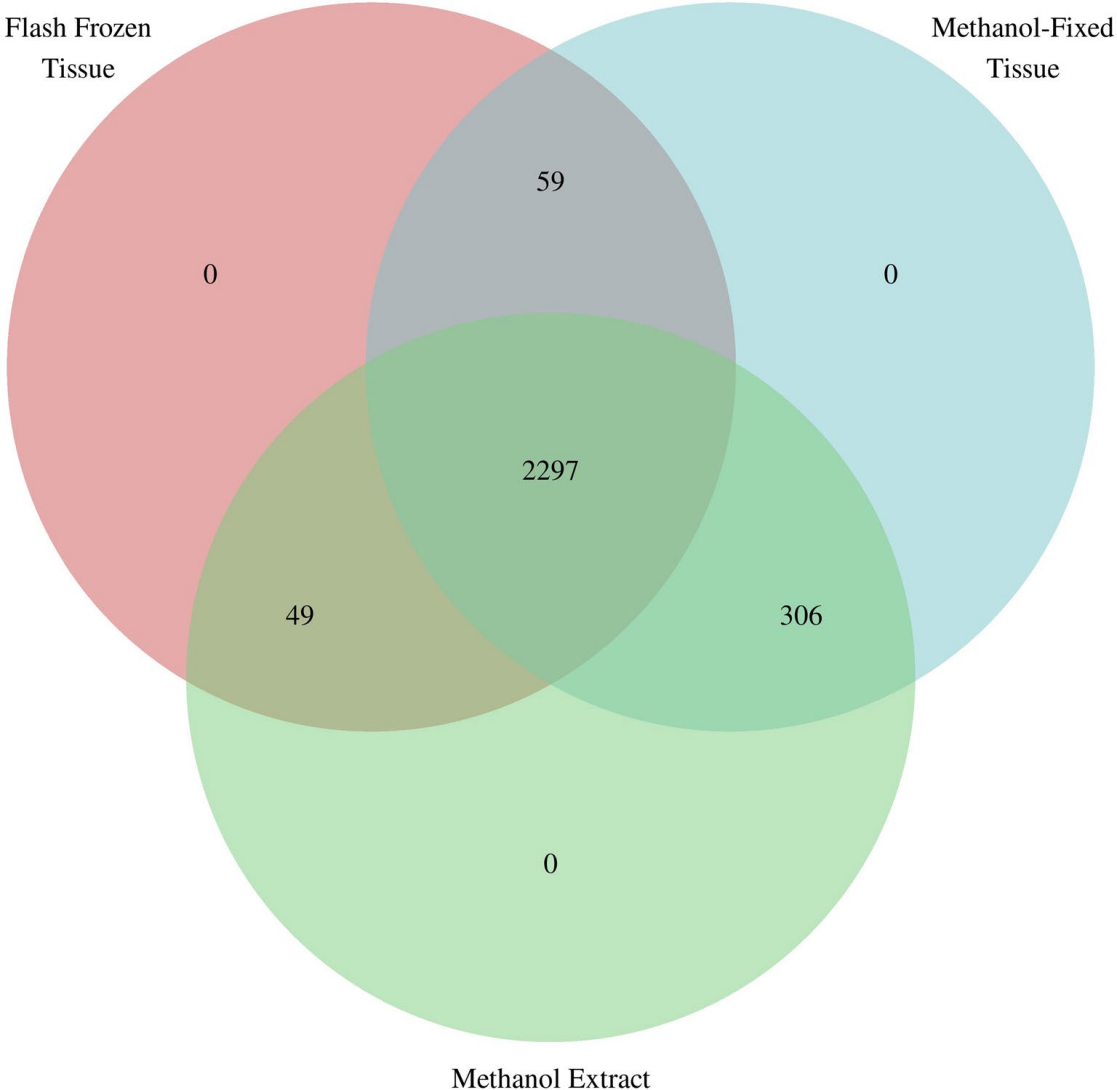
Number of metabolite features shared across the three sample types (flash frozen tissue, methanol-fixed tissue, and methanol extract).

Figure 4 presents the number of metabolites that were in the flash frozen samples were also in the methanol-extract and the methanol-fixed tissue samples. Of the 2405 metabolite features detected in at least 2/3rds of flash frozen samples, 2356 (98%) were also detected in at least 2/3rds of methanol-fixed tissue samples. Further, 2346 (98%) were also detected in at least 2/3rds of methanol extract samples. There were 306 metabolite features found only in the methanol samples and not in the flash frozen samples.

## Discussion

This study provides data comparing methanol fixation, a method that does not require immediate flash-freezing of specimens, with flash-freezing for the measurement of placental metabolomics. Results demonstrate that placental tissue samples collected in 80% methanol at room temperature can yield similar metabolic data to flash frozen specimens. In general, the clustering of the flash-frozen and methanol-treated tissue profiles revealed by PCA, when taken with the Venn diagram results, suggest that the overwhelming majority of metabolites were preserved with methanol fixation. The duration of fixation in 80% methanol was less impactful than sample type with only about a fifth of metabolites displaying a significant time trend, and the direction of association was metabolite-specific. While profiles of placenta tissue fixed in methanol were similar to those of flash frozen samples, the metabolic profile of methanol extract samples differed from the gold standard of flash freezing, having more metabolite features detected as well as significantly higher intensities that explained a large proportion of the variability. The high RSD suggests that results are reproducible.

Additional work is needed to characterize the differences between metabolites in the methanol extract and the methanol-fixed tissue. While we expect that some metabolites will leach out of the tissue and become suspended in the methanol, it was surprising that higher intensities were observed for metabolites in the methanol extract. Sauerschnig et al. reported that extraction of metabolites with methanol can result in creation of artifacts^14^. In the present study we removed all metabolites that were found in blank methanol control samples as these were likely contaminants and artifacts, nonetheless, residual artifacts remain a possibility. These findings emphasize the importance of running blank methanol control samples as a quality assurance step in future studies. Finally, it remains possible that there was incomplete quenching by the methanol, though this seems unlikely as we would have expected a greater number of different metabolites between the flash frozen and methanol fixed samples. Future work will delineate which metabolites display higher intensities.

Some limitations of this method merit mention. Of note, 80% methanol is flammable and can be toxic to humans^16^. As such, it is best used in small volumes under controlled environments and under a fume hood. For the samples collected in this study, less than 2 mL of 80% methanol were used in the collection of each placental biospecimen. The risks and benefits and availability of a fume hood should be considered when deciding to utilize this approach.

In summary, this work suggests that collection of specimens at room temperature in 80% methanol is a viable method for metabolic profiling, and that tissue fixed in methanol for 1-h, 12-h, and 48-h did not have significantly more metabolites detected compared to the flash frozen specimens. Given the similarity between flash frozen tissue and tissue collected and fixed in methanol for 1-h (as well as the logistical ease of shorter fixation times), this sample type is our general recommendation for an alternative approach to flash freezing for studies focused on metabolomics. In many cases, investigators will have to decide whether to analyze methanol-fixed tissue or the methanol extract. While results suggest that similar metabolites are detected in both specimens (methanol fixed tissue and the methanol extract), the higher intensities in the methanol extracts may allow for the discovery of new metabolites or observation of metabolites found at low intensities using flash frozen samples. It might also preserve tissue for other analyses such as transcriptomics as methanol fixation preserves tissue DNA and RNA^17,18^. On the other hand, it renders comparison to previously published findings potentially more difficult. As such, the decision on what to analyze for metabolites is ultimately project and goal specific. Regardless, this methodology allows for the collection of tissue samples at room temperature, which is more feasible to do in community-based hospitals.

## Supplementary Information

Supplementary Table S1.

Supplementary Table S2.

## Data Availability

The datasets analyzed for the current study are available from the corresponding author on reasonable request.
