# Supplementary material for: Comparison of methanol fixation versus cryopreservation of the placenta for metabolomics analysis
Source: Sci Rep. 2023 Mar 11;13:4063. doi: 10.1038/s41598-023-31287-3 (PMC10008642; doi:10.1038/s41598-023-31287-3)
Supplement: Supplementary file 1 — Supplementary Table S1. [file 41598_2023_31287_MOESM1_ESM.docx]

**Supplemental Table 1**. List of internal standards.

| **Compound** |
| --- |
| ^13^C_3_ Lactate |
| ^13^C_5_ alpha-ketoglutarate |
| ^13^C_6_ citrate |
| ^13^C_4_ succinate |
| ^13^C_4_ malate |
| U-^13^C amino acid mix (13C-algal, Sigma) |
| U-13C glutamine |
| U-13C cystine |
| 15N asparagine |
| 15N2 Tryptophan |
| ^13^C_6_Glucose |
| D4-thimine |
| 15N anthranillic acid |
| Gibberelic acid |
| Epibrassinolide |
